# Multi-feature fusion network with marginal focal dice loss for multi-label therapeutic peptide prediction

**DOI:** 10.1371/journal.pcbi.1013622

**Published:** 2025-10-27

**Authors:** Yijun Mao, Yurong Weng, Jian Weng, Ming Li, Wanrong Gu, Rui Pang, Xudong Lin, Yunyan Xiong, Deyu Tang

**Affiliations:** 1 College of Mathematics and Informatics, South China Agricultural University, GuangZhou, GuangDong, China; 2 National Key Laboratory of Data Space Technology and System, Beijing, China; 3 College of Cyber Security, Jinan University, GuangZhou, GuangDong, China; 4 College of Plant Protection, South China Agricultural University, GuangZhou, GuangDong, China; 5 School of Computer and Information Engineering, Guangdong Polytechnic of Industry and Commerce, GuangZhou, GuangDong, China; Zhejiang University of Technology, CHINA

## Abstract

Accurately predicting the functions of multi-functional therapeutic peptides is crucial for the development of related drugs. However, existing peptide function prediction methods largely rely on either a single type of feature or a single model architecture, limiting prediction accuracy and applicability. Additionally, training better-performing models on datasets with class imbalance issues remains a significant challenge. In this study, we propose the multi-functional therapeutic peptide of multi-feature fusion prediction (MFTP_MFFP) model, a novel method for predicting the functionality of multi-functional therapeutic peptides. This approach uses various encoding techniques to process peptide sequence data, generating multiple features that help the model learn hidden information within the sequences. To maximize the effectiveness of these features, we propose a gated feature fusion module that efficiently integrates them. The module assigns learnable gating weights to each feature, optimizing integration and enhancing fusion efficiency. The fused features are then passed into a neural network model for feature extraction. Additionally, we propose a marginal focal dice loss function (MFDL) to address the class imbalance and improve the model’s prediction performance. Experimental results show that the MFTP_MFFP model outperforms existing models in all evaluation metrics, demonstrating its robustness and effectiveness in multi-functional therapeutic peptide prediction tasks.

## Author summary

Understanding the biological complexity and sequence ambiguity of multi-functional therapeutic peptides (MFTPs) is crucial for developing robust prediction models. In this work, we present MFTP_MFFP, a unified deep learning framework that combines biological domain knowledge with adaptive architectural strategies. Unlike prior methods that rely on fixed structures or single-source encodings, our approach integrates diverse biological features—including statistical, physicochemical, and evolutionary encodings—processed through fuzzy transformation to enhance robustness against uncertainty. By incorporating graph-based modeling and gated feature fusion, the framework captures both sequential and topological signals. Rather than deepening the network blindly, we emphasize structural adaptability and biological relevance. We hope this work offers insights into feature fusion, imbalance-aware loss design, and evolutionary optimization for future peptide-based prediction tasks.

## Introduction

In recent years, bioactive peptides have drawn increasing attention due to their promising therapeutic applications and continuous advancements in peptide synthesis and analytical techniques [1]. Among these, MFTPs, characterized by short amino acid (AA) sequences, have emerged as particularly valuable due to their diverse biological activities. These peptides play critical roles across multiple disciplines, including agriculture, medicine, and microbiology, functioning effectively as hormones, growth factors, neurotransmitters, ion channel ligands, and anti-infective agents, among others [2]. The growing recognition of their versatile functions underscores the need for accurate and reliable methods for predicting their biological roles.

To advance peptide research, substantial efforts have been made toward building comprehensive peptide sequence databases [3–5]. Concurrently, machine learning has emerged as a powerful approach for predicting peptide functions, uncovering novel insights into their diverse biological activities. However, existing computational methods still face notable limitations. For instance, Xu et al. proposed a convolutional neural network (CNN)-based method achieving commendable performance, yet the method inadequately utilized structural and positional details of amino acids within peptide sequences, potentially limiting prediction accuracy for specific functional categories [6]. Yan et al. developed PrMFTP, a multi-label predictor employing deep neural networks (DNN). Despite this innovation, their model did not sufficiently incorporate feature preprocessing techniques to enhance robustness during training [7]. Furthermore, Fan et al. introduced a multi-label focal dice loss (MLFDL), combining Focal Loss [8] and Dice Loss [9], alongside text convolution techniques. Nevertheless, their method treated peptide sequences as plain linear strings, overlooking the underlying physicochemical characteristics that are essential for accurately capturing their biological functions [10].

Additionally, severe class imbalance commonly encountered in peptide datasets remains inadequately addressed by traditional loss functions, causing models to disproportionately focus on majority classes while failing to accurately predict minority classes, which often represent biologically significant but rare functionalities. Although previous studies attempted to alleviate class imbalance, insufficient attention was given to effectively emphasizing minority classes, thus highlighting a critical gap that needs to be addressed to improve overall prediction accuracy and reliability.

To address the limitations of existing methods, we propose a novel predictive framework named MFTP_MFFP. This model introduces several key innovations aimed at enhancing the representation and interpretation of peptide sequences. We employ five complementary biological feature encoding strategies to capture diverse physicochemical and functional characteristics of amino acids. These encodings are further refined through fuzzification techniques, which help the model handle uncertainty and biological ambiguity more effectively, thereby improving its robustness and generalization ability. To better represent the spatial structure of peptide sequences, we transform them into adjacency matrices and use Graph Attention Convolution (GATConv) [11] to extract refined positional features, capturing the spatial relationships between residues that are often overlooked by traditional sequence-based models. In addition, we design a gated feature fusion module to integrate these heterogeneous features, allowing the model to adaptively weigh the importance of different feature sources and achieve consistent and efficient feature aggregation. Finally, to alleviate the class imbalance problem commonly found in peptide datasets, we introduce a novel loss function—MFDL—which combines the strengths of Label-Distribution-Aware Margin (LDAM) [12] and MLFDL. This function dynamically adjusts margins for each sample and reweights difficult instances, thereby strengthening the model’s ability to recognize underrepresented functional classes and improving overall prediction performance.

## Results

### Different multi label prediction models

We present four CNN-based multi-label prediction models designed to address the complexities inherent in multi-label prediction tasks. These models improve prediction accuracy and robustness through diverse architectural designs and advanced feature extraction techniques. To ensure fair performance evaluation, a five-fold cross-validation method was employed [13]. All models were trained and evaluated on the same dataset to maintain consistency and comparability. Detailed performance metrics for each model on the test set are summarized in Table 1. Experimental results indicate that the MFTP_MFFP model consistently outperforms other models during the testing phases, delivering superior results in both prediction accuracy and robustness. The MFTP_MFFP model integrates BiLSTM [14,15] and CNN layers with various feature types, allowing for a more comprehensive understanding of sequence information. Furthermore, the gate feature fusion module is utilized to seamlessly integrate information from multiple sources, thereby enhancing decision-making accuracy.

**Table 1:** The performance of different multi-label prediction models for MFTP prediction.^a^

Model	Precision	Coverage	Accuracy	Absolute true	Absolute false	F1-score	MCC
CNN	0.421±0.012	0.416±0.013	0.391±0.015	0.344±0.014	0.066±0.002	0.400±0.006	0.381±0.011
CNN+GATConv	0.418±0.014	0.416±0.013	0.388±0.014	0.339±0.011	0.067±0.001	0.389±0.007	0.377±0.012
CNN+BiLSTM	0.410±0.012	0.407±0.012	0.381±0.009	0.337±0.013	0.069±0.005	0.373±0.010	0.351±0.08
CNN+Transformer	0.659±0.010	0.649±0.008	0.614±0.012	0.543±0.011	0.043±0.003	0.609±0.006	0.600±0.006
CNN+GMF	0.695±0.013	0.687±0.011	0.648±0.010	0.575±0.012	0.039±0.003	0.657±0.008	0.633±0.011
**MFTP_MFFP**	**0.729± 0.010**	**0.728± 0.009**	**0.688± 0.011**	**0.615± 0.012**	**0.036± 0.002**	**0.685± 0.013**	**0.665± 0.012**

^a^ The best values are highlighted in bold.

In conclusion, the MFTP_MFFP model’s innovative architecture and advanced feature extraction techniques enable it to effectively manage the complexities of multi-label prediction tasks, achieving superior performance in both training and testing phases.

### Ablation analysis

We conducted an extensive ablation study to comprehensively evaluate the importance of each component within the MFTP_MFFP model. The ablation study consisted of two parts: module ablation and feature ablation. The results of the ablation experiments are summarized in Table 2. The primary objective of this analysis was to assess the independent contribution of each module and feature to the overall model performance. By systematically removing or modifying specific modules and features, we aimed to clarify their impact on the model architecture. To ensure robust and reliable results, a five-fold cross-validation approach was adopted. First, the complete MFTP_MFFP model, which included all designed modules, was used as the baseline model for performance comparison. Multiple model variants were then constructed, with each variant removing a specific module or feature. All other hyperparameters were kept constant to ensure that any observed differences in performance could be attributed solely to the absence of the corresponding module or feature. For the module ablation, all modules except for the gated feature fusion module were directly removed. As the gated feature fusion module involves the integration of multiple features, we replaced it by simply summing features after aligning their dimensions. For the feature ablation, we assigned zeros to the ablated feature to measure its contribution to model performance.

**Table 2:** The performance of MFTP_MFFP and its variants on the test set.^a^

Model	Precision	Coverage	Accuracy	Absolute true	Absolute false	F1-score	MCC
No GATConv	0.702±0.013	0.706±0.012	0.658±0.016	0.580±0.014	0.038±0.003	0.677±0.013	0.653±0.010
No BiLSTM	0.700±0.015	0.700±0.011	0.655±0.011	0.579±0.012	0.039±0.002	0.668±0.011	0.640±0.009
No GMF	0.716±0.005	0.713±0.010	0.672±0.08	0.601±0.007	0.038±0.001	0.671±0.010	0.653±0.008
No Gate feature fusion	0.707±0.012	0.695±0.012	0.653±0.013	0.566±0.011	0.036±0.003	0.663±0.012	0.651±0.011
No CNN	0.592±0.010	0.581±0.013	0.548±0.009	0.481±0.011	0.045±0.003	0.584±0.011	0.561±0.008
No FFN	0.713±0.008	0.708±0.014	0.670±0.011	0.600±0.011	0.037±0.002	0.666±0.008	0.645±0.010
No Embedding encode	0.610±0.012	0.619±0.010	0.573±0.009	0.499±0.013	0.047±0.001	0.592±0.012	0.567±0.011
No Attention encode	0.685±0.011	0.683±0.012	0.641±0.013	0.562±0.007	0.040±0.001	0.652±0.010	0.631±0.013
No AAIndex encode	0.715±0.007	0.714±0.010	0.672±0.011	0.598±0.011	0.037±0.003	0.674±0.013	0.658±0.011
No PAAC encode	0.714±0.008	0.713±0.012	0.673±0.009	0.602±0.010	0.038±0.005	0.672±0.011	0.652±0.014
No PC6 encode	0.708±0.013	0.700±0.012	0.665±0.014	0.598±0.006	0.038±0.002	0.666±0.005	0.646±0.010
No BLOSUM62 encode	0.690±0.015	0.688±0.011	0.646±0.012	0.570±0.012	0.040±0.003	0.651±0.009	0.630±0.010
No AAC encode	0.682±0.013	0.680±0.007	0.637±0.013	0.562±0.011	0.041±0.003	0.641±0.011	0.619±0.015
No Graph-based encode	0.694±0.012	0.687±0.011	0.649±0.012	0.576±0.09	0.039±0.003	0.654±0.010	0.633±0.011
**MFTP_MFFP**	**0.729± 0.010**	**0.728± 0.009**	**0.688± 0.011**	**0.615± 0.012**	**0.036± 0.002**	**0.685± 0.013**	**0.665± 0.012**

^a^ The best values are highlighted in bold. The principles and functions of each module mentioned in the table, based on the just-in-time coding approach, will be elaborated in the Materials and methods section below.

Specifically, at the module level, GAT facilitates the effective extraction of positional information from graph-structured data; BiLSTM captures contextual dependencies at each position in the sequence; GMF enhances model robustness by fuzzifying biological features using Gaussian membership functions; and the gated feature fusion module optimizes the integration of multiple features through learnable gating weights. At the feature level, the Embedding encoder provides fundamental structural information for downstream modeling; the Attention encoder leverages multi-head attention to capture long-range residue interactions; the AAIndex [16] encoding provides the physicochemical properties of amino acids, enhancing the model’s ability to generalize to diverse sequences; PAAC focuses on the micro-arrangement of sequences, which is crucial for distinguishing peptides with similar compositions but different functions; PC6 encoding provides a compact and effective biochemical perspective, particularly valuable when parameters or samples are limited; BLOSUM62 encoding helps identify conserved yet functionally critical residue fragments; AAC [17] encoding captures the overall biological preference of peptides; and graph-based encoding captures the structural relationships between local fragments. Notably, removing any single module or feature consistently resulted in a drop in multiple performance metrics, indicating that each module and feature contributes positively to the overall performance of the model.

In conclusion, the ablation study provides strong evidence for the interdependence and synergy among the modules and features within the MFTP_MFFP model.

### Performance comparison of different loss functions

Class imbalance is a common and persistent challenge in multi-label prediction tasks, where certain functional categories are significantly underrepresented compared to others. This imbalance often distorts the learning process, causing traditional loss functions to be overly biased toward majority classes and resulting in suboptimal recognition of rare categories. As a consequence, models trained with standard objective functions may achieve high overall accuracy while failing to effectively capture critical minority class information.

To address this issue, we propose MFDL, a loss function specifically designed to enhance the learning of minority classes during training. MFDL integrates two key strategies: (1) assigning higher weights to underrepresented classes to counteract their low frequency, and (2) introducing margin-based adjustments inspired by label-distribution-aware methods to effectively enlarge the decision boundary margins for minority categories. This dual strategy encourages the model to focus more on difficult or rare labels without sacrificing overall prediction stability or increasing the risk of overfitting.

To rigorously evaluate the effectiveness of MFDL, we conducted comparative experiments against several widely used loss functions, including AsymmetricLoss [18], BCEWithLogitsLoss, MLFDL, and LDAM. All experiments followed a standardized five-fold cross-validation protocol to ensure statistical robustness and minimize sampling bias. Each model variant maintained identical architectures and training procedures to isolate the impact of the loss function. The comparative results summarized in Table 3 show that the MFTP_MFFP model trained with MFDL consistently outperformed other variants across most major evaluation metrics. Although LDAM achieved slightly higher coverage, MFDL delivered a more balanced performance, effectively improving minority class recognition while maintaining competitive overall metrics.

**Table 3:** Performance comparison of MFTP_MFFP with different loss functions on the test set.^a^

Model	Precision	Coverage	Accuracy	Absolute true	Absolute false	F1-score	MCC
AsymmetricLoss	0.697±0.006	0.725±0.003	0.659±0.003	0.572±0.005	0.041±0.002	0.649±0.007	0.628±0.010
BCEWithLogitsLoss	0.706±0.005	0.703±0.011	0.659±0.004	0.586±0.007	0.037±0.002	0.672±0.008	0.650±0.009
MLFDL	0.705±0.010	0.706±0.009	0.662±0.007	0.587±0.004	0.039±0.001	0.673±0.008	0.655±0.004
LDAM	0.679±0.012	**0.743± 0.012**	0.648±0.010	0.540±0.008	0.044±0.002	0.670±0.009	0.649±0.012
FocalLoss	0.616±0.011	0.721±0.008	0.596±0.006	0.457±0.005	0.045±0.002	0.636±0.011	0.601±0.008
**MFDL**	**0.729± 0.010**	0.728±0.009	**0.688± 0.011**	**0.615± 0.012**	**0.036± 0.002**	**0.685± 0.013**	**0.665± 0.012**

^a^ The best values are highlighted in bold.

**Fig 1:**
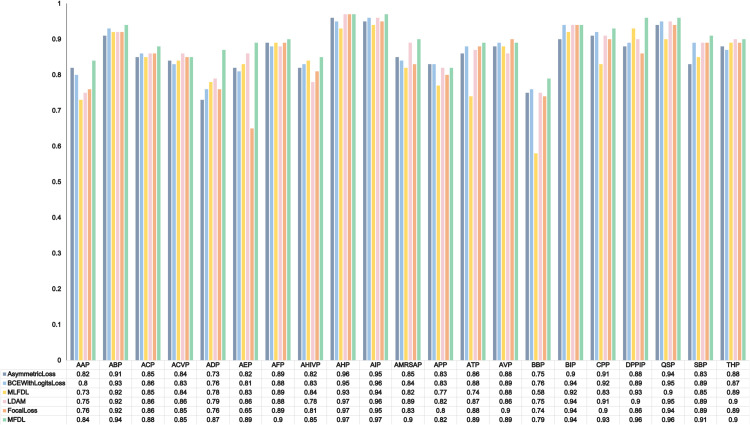
AUC values of 21 functions in different loss functions.

To further validate the superiority of MFDL, particularly its performance on individual categories, we plotted ROC curves for each functional category and used the area under the curve (AUC) as an overall performance metric to evaluate the model’s classification ability in each category. This approach enables a more direct assessment of the effectiveness of the loss function. Given the large number of categories, we extracted the AUC values from the ROC curves and presented them as bar charts to provide a more intuitive illustration of the model’s performance on low-frequency categories. These results are shown in Fig 1. The results demonstrate that the model trained with MFDL achieved the highest AUC in most categories, further confirming the effectiveness of MFDL in mitigating imbalance-related biases and enabling more comprehensive and reliable functional peptide prediction.

### Performance comparison of MFTP_MFFP with the existing methods

To further validate the effectiveness of the MFTP_MFFP model, we conducted comparative experiments against several state-of-the-art methods for amino acid sequence function prediction. The selected baselines include MPMABP [19], MLBP [20], SP-RNN [21], PrMFTP, ETFC, and MFTP-TOOL [22], all of which represent strong benchmarks in the domain. These comparisons offer a comprehensive assessment of MFTP_MFFP’s capabilities relative to leading approaches.

To ensure a fair and consistent comparison, all models were trained and evaluated on the same dataset, using identical training splits and preprocessing pipelines. To further minimize the influence of random sampling and enhance robustness, we randomly selected 80% of the original test set to construct five distinct validation subsets. Each model was evaluated on these subsets, and the results were averaged to obtain reliable estimates of performance across all metrics. As summarized in Table 4, the MFTP_MFFP model consistently outperformed all other baseline methods across key evaluation metrics. The observed improvements can be attributed to MFTP_MFFP’s unique design, which integrates diverse feature encoding strategies and advanced neural network components. Unlike conventional models, MFTP_MFFP extracts comprehensive sequence-level representations through a combination of BiLSTM, CNN, GATConv, and a gated fusion module. The incorporation of MFDL also enhances the model’s ability to detect underrepresented classes by effectively addressing class imbalance.

**Table 4:** Performance comparison of MFTP_MFFP with existing models.^a^

Model	Precision	Coverage	Accuracy	Absolute true	Absolute false	F1-score	MCC
MPMABP	0.373±0.008	0.351±0.010	0.340±0.011	0.307±0.007	0.043±0.002	0.335±0.012	0.317±0.010
MLBP	0.588±0.010	0.594±0.011	0.550±0.013	0.476±0.012	0.040±0.002	0.530±0.09	0.521±0.013
SP-RNN	0.604±0.008	0.620±0.012	0.566±0.008	0.482±0.010	0.038±0.002	0.548±0.015	0.524±0.012
PrMFTP	0.650±0.013	0.650±0.015	0.609±0.012	0.536±0.013	0.038±0.003	0.611±0.011	0.603±0.010
ETFC	0.709±0.011	0.710±0.012	0.669±0.010	0.598±0.008	0.037±0.002	0.676±0.006	0.633±0.006
MFTP-TOOL	0.710±0.011	0.721±0.009	0.676±0.008	0.585±0.012	0.036±0.002	0.673±0.008	0.651±0.011
**MFTP_MFFP**	**0.729± 0.010**	**0.728± 0.009**	**0.688± 0.011**	**0.615± 0.012**	**0.036± 0.002**	**0.685± 0.013**	**0.665± 0.012**

^a^ The best values are highlighted in bold.

Overall, these results demonstrate that MFTP_MFFP achieves superior predictive performance compared to established methods. Its architectural innovations and robust learning strategy position it as a powerful tool for multi-label amino acid sequence functionality prediction.

## Discussion

In this study, we propose the MFTP_MFFP model, a novel deep learning architecture specifically tailored for predicting the functionality of multifunctional therapeutic peptides (MFTPs). This model integrates several key innovations, including the Margin-Focal Dice Loss (MFDL) function designed to mitigate the effects of severe class imbalance, and a gated feature fusion module that dynamically weights and integrates multiple heterogeneous feature types. Additionally, the model incorporates graph attention convolution to effectively capture residue-level interactions and topological patterns, along with multiple biologically relevant sequence encodings and a feature fuzzification layer to enhance representation flexibility and robustness.

Extensive experiments demonstrate that the MFTP_MFFP model exhibits a strong capacity to capture complex functional and structural patterns embedded in peptide sequences, outperforming existing methods across multiple evaluation metrics. The MFDL component contributes to a more balanced training process, boosting the recognition of underrepresented peptide categories, while the fusion module ensures that complementary information from diverse sources is optimally leveraged.

We believe the MFTP_MFFP model holds great promise as an effective computational tool for accelerating MFTP discovery and characterization. In real-world scenarios, the model can be applied to pre-screen large-scale peptide libraries to identify multifunctional candidates—such as peptides with both anticancer and antimicrobial activity—thus substantially reducing the experimental workload. This pre-screening step can streamline the drug development pipeline by prioritizing peptides for downstream synthesis, in vitro and in vivo validation. Furthermore, the model can be embedded into peptide engineering workflows, facilitating the rational design of novel therapeutic agents and lowering the costs and time associated with early-stage drug discovery.

In summary, the proposed MFTP_MFFP model achieved strong performance in predicting multifunctional therapeutic peptides, validating the effectiveness of the proposed methods. This study provides a feasible computational approach for peptide function screening and offers practical experience for applying deep learning techniques to biological sequence analysis.

## Materials and methods

### Dataset

In this study, we utilized the benchmark dataset from the PrMFTP model to train and comprehensively evaluate the performance of our proposed MFTP_MFFP model. The dataset contains 9,874 peptide amino acid sequences, which were randomly divided into a training set (80%) and an independent testing set (20%). The distribution of single-functional and multi-functional therapeutic peptides in both sets is shown in Table 5.

**Table 5:** Distribution of single-functional and multi-functional therapeutic peptides in the training and testing sets.

Type	Train	Test
Single-functional therapeutic peptides	6714	1697
Multi-functional therapeutic peptides	1185	278

**Fig 2:**
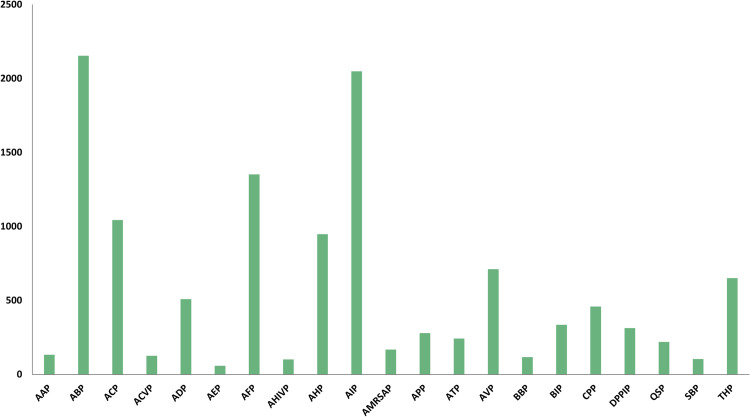
Distribution of therapeutic peptides across functional categories in the dataset.

Fig 2 illustrates the number of therapeutic peptides assigned to each functional category. For multi-functional peptides, each annotated function contributes to the corresponding category’s count. The dataset encompasses 21 distinct functional classes, covering a wide range of biological activities, including: anti-microbial peptides (AMPs), anti-bacterial peptides (ABPs), anti-cancer peptides (ACPs), anti-coronavirus peptides (ACVPs), anti-diabetic peptides (ADPs), anti-endotoxin peptides (AEPs), anti-fungal peptides (AFPs), anti-HIV peptides (AHIVPs), anti-hypertensive peptides (AHPs), anti-inflammatory peptides (AIPs), anti-MRSA peptides (AMRSAPs), anti-parasitic peptides (APPs), anti-tubercular peptides (ATPs), anti-viral peptides (AVPs), blood-brain barrier peptides (BBPs), biofilm-inhibitory peptides (BIPs), cell-penetrating peptides (CPPs), dipeptidyl peptidase IV peptides (DPPIPs), quorum-sensing peptides (QSPs), surface-binding peptides (SBPs), and tumor homing peptides (THPs).

### Method framework

**Fig 3:**
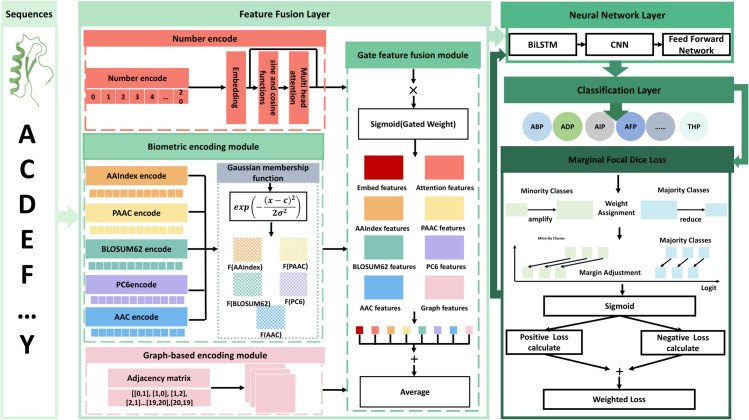
The architecture of MFTP_MFFP. First, the model processes amino acid sequences through three encoding strategies: numerical encoding, biological feature encoding, and graph-based encoding. Numerical encoding generates high-dimensional vectors via embedding layers and position-aware transformations; biological encoding applies five feature extraction methods combined with Gaussian fuzzification; graph-based encoding captures spatial relationships through graph neural networks. Together, these produce eight distinct feature sets. These features are fused using a gated feature fusion module with learnable dynamic weights. Finally, the fused representation is processed by a neural network comprising BiLSTM, CNN, and feedforward layers to generate functional predictions. The propagation process of the loss function is also shown in the figure. The F(AAindex), F(AAC), F(BLOSUM62), F(PC6), and F(AAC) denote the fuzzified biological features.

We propose the MFTP_MFFP model for the prediction of MFTPS, which is structured into three primary layers: the feature fusion layer, the neural network layer, and the classification layer. The overall architecture of the model is depicted in Fig 3. To facilitate a comprehensive understanding, the specific roles and operational mechanisms of each layer are described in detail in the following sections.

#### Feature fusion layer.

To effectively integrate the diverse information derived from peptide sequences, we construct a multi-component Feature Fusion Layer as the foundational module of our architecture. Rather than directly merging raw inputs, this layer performs shallow feature extraction and alignment across heterogeneous feature representations derived from various encoding strategies. These include transformations such as embedding layers, linear projections, fuzzy mappings, and graph-based encodings, which help standardize different input modalities into a unified representation space. This layer serves as a preprocessing and integration hub, focusing on modality-specific encoding and adaptive feature fusion. Deeper semantic modeling—such as capturing temporal dependencies and hierarchical patterns—is performed by the downstream neural network layers. The Feature Fusion Layer is composed of four functional submodules: a numerical encoding module, a biological encoding module, a graph-based encoding module, and a gated feature fusion module. The first three handle domain-specific transformations, while the gated module adaptively integrates the outputs via a learned weighting mechanism. A detailed description of each module is presented in the following sections.

**Digital encoding module.** The peptide AA sequences are first subjected to numerical encoding and padding. Each amino acid is assigned an integer between 1 and 20 based on its alphabetical order among the 20 standard amino acids (A, C, D, E, F, ..., Y). Considering that the sequence lengths in the benchmark dataset range from 5 to 50, sequences shorter than 50 amino acids are padded with zeros to maintain a consistent input length. In addition, to prevent the padded zeros from interfering with feature extraction, we explicitly apply mask processing to the padded positions during subsequent feature learning stages, ensuring that the neural network focuses solely on the true amino acid information. This design enhances the accuracy of feature learning and improves the model’s generalization capability.

The numerically encoded and padded sequences are then passed through an embedding layer, which projects the fixed-length sequences (50 AAs) into a high-dimensional vector space, forming the first representation feature. We adopt sine and cosine functions to incorporate positional information into this feature, following the positional encoding method proposed by Vaswani et al. [23] and further extended by Chu et al. [24]. These encodings represent the relative positions of amino acids in the sequence, and the corresponding equations used for generation are as follows:

$$PE_{\left(pos,2i\right)}=\sin\left(\frac{pos}{10000^{\frac{2i}{d_{\text{model}}}}}\right)$$ (1)

$$PE_{\left(pos,2i+1\right)}=\cos\left(\frac{pos}{10000^{\frac{2i}{d_{\text{model}}}}}\right)$$ (2)

where *PE*_(*pos*,2*i*)_ represents the value computed for position *pos* in the 2*i*-th dimension using a sine function, while *PE*_(*pos*,2*i* + 1)_ represents the value for position *pos* in the 2i+1-th dimension using a cosine function. The parameter $d_{\text{model}}$, referring to the total dimensionality of the encoding vector, is carefully selected to facilitate subsequent processing stages. The vectors derived from sine and cosine functions are input into the MHSA for further processing, and the resulting outputs are treated as the second feature.

**Biometric encoding module.** Numerical encoding is a commonly used initial method for peptide representation; however, it lacks the capacity to reflect the complex biological and structural information inherent in AA sequences. To address this, we complement the numerical encoding with five biologically informed feature encoding methods that reflect diverse physicochemical, biochemical, and evolutionary properties. These include AAIndex, PAAC, PC6, BLOSUM62, and AAC encoding.

The AAIndex encoding maps each AA to a 531-dimensional vector derived from curated physicochemical and biochemical indices, enriching the sequence with detailed descriptors such as hydrophobicity and charge. PAAC and PC6 compress these properties into lower-dimensional representations, capturing core features like hydrophilicity, side chain mass, polarity, and molecular weight in 3- and 6-dimensional formats, respectively. These methods help identify subtle structural variations and potential functional regions. BLOSUM62, based on substitution probabilities, highlights conserved and functionally critical residues through a 23-dimensional mapping. In contrast, AAC abstracts the global composition of the sequence into a frequency-based 20-dimensional vector, offering a holistic perspective on sequence characteristics. By combining these complementary encodings, the model gains a multifaceted understanding of peptide sequences—from local chemistry to global structure, from evolutionary conservation to residue-level detail. This multi-view representation lays a biologically rich foundation for downstream prediction.

To further improve robustness under noisy or ambiguous inputs, we introduce a fuzzification layer inspired by fuzzy neural networks [25]. This layer applies Gaussian membership functions (GMF) [26] to transform crisp feature values into soft membership degrees. By blurring the boundaries of feature values, the fuzzification layer enhances noise tolerance and generalization capability. It enables the model to better interpret ambiguous inputs and flexibly capture latent biological variation. The GMF formula is defined as follows:

$$f(x)=\exp\left(-\frac{(x-c{)}^{2}}{2\sigma^{2}}\right)$$ (3)

where *x* is the input feature , *f*(*x*) is the fuzzy output of the input *x*, *c* is the center (mean) of the GMF, and σ is the standard deviation, controlling the spread or fuzziness of the membership.

**Graph-based encoding module.** The positional relationships AAs within peptide sequences play a fundamental role in shaping their biological functions and structural organization, impacting the formation of functional sites, the stabilization of three-dimensional structures, and interactions with other biomolecules [27–31]. While basic positional information is embedded during the numerical encoding step via sinusoidal functions, these methods inherently treat residues in isolation, lacking the capacity to dynamically model inter-residue dependencies based on the sequence context.

To address these limitations, we construct an explicit graph-based representation of peptide sequences in our framework. In this representation, each amino acid is modeled as a node, and undirected edges are established between sequentially adjacent residues, thereby preserving the natural topology of the peptide backbone. Specifically, in our implementation, each residue at position *i* is connected bidirectionally to residues at positions *i*–1 and i+1 where available, and the graph is encoded as an adjacency matrix and fed into a graph neural network (GNN) module. Although such a linear graph structure might initially appear redundant given the sequential nature of peptides, modeling peptides as graphs introduces important inductive biases that can be exploited by Graph Neural Networks (GNNs) [32]. In particular, the use of GATConv allows the model to dynamically modulate the strength of interactions between neighboring residues. Instead of treating all sequential neighbors equally, the GATConv architecture employs attention mechanisms to learn the functional relevance of each connection, enabling the model to prioritize contextually important interactions while down-weighting less informative ones.

Moreover, because GATConv propagates information across the graph structure iteratively, this architecture enables the capture of not only immediate sequential dependencies but also higher-order, long-range relationships between residues. This dynamic and hierarchical modeling approach enriches the representation space of peptides, allowing the network to identify functional motifs, recognize critical residue clusters, and infer complex dependency patterns that may not be evident through static feature encodings alone.

**Gate feature fusion module.** Finally, by integrating features extracted from numerical, biological, and graph-based encodings, our model generates eight heterogeneous feature streams, each capturing complementary aspects of peptide sequences, including positional, structural, and biochemical information. Effectively fusing such diverse representations remains a non-trivial task, as conventional fusion strategies often fail to capture the nuanced interactions among modalities with differing statistical properties and biological relevance. To address this, we introduce a gated feature fusion module, a central component of our architecture that enables dynamic and learnable integration of multi-source features. Instead of treating all feature streams equally, the proposed method assigns each modality a trainable gating weight, which regulates its relative influence in the final fused representation. These weights are learned end-to-end during model training and allow the network to automatically emphasize more informative modalities while attenuating less relevant ones.

Gated Weight Initialization: At the start of training, the fusion module initializes a gating vector for each feature stream. The dimensions of each vector are aligned with its respective input, and all gating vectors are initialized randomly. During training, these weights are updated via backpropagation, enabling the model to iteratively refine the relative contributions of each feature source. This adaptive fusion mechanism ensures that the final representation optimally reflects the most salient features, thereby improving performance on downstream predictive tasks. Assume there are *N* feature sources, each with dimension *d*, *b* is the batch size of the data, and *l* is the length of the sequences. For each feature source *i*, let ${𝐅}_{i}\in {\mathbb{R}}^{\text{b}\times \text{l}\times d}$ denote the feature representation. The gate weight matrix 𝐆 is initialized as a matrix of size N×d, where each row ${𝐠}_{i}\in {\mathbb{R}}^{d}$ represents the gating weight vector for the *i*-th feature source.

During forward propagation, each input feature stream is modulated by a gating weight that is passed through a sigmoid activation function to constrain its value within the range [0, 1] [33]. This bounded, continuous weight enables the model to assign differentiated importance levels to each feature source in a smooth and trainable manner. By applying the sigmoid function, the network adaptively amplifies or suppresses individual features based on learned relevance during training. The mathematical formulation of the gating mechanism is given as follows:

$${\tilde{𝐅}}_{i}={𝐅}_{i}⊙\sigma ({𝐠}_{i})$$ (4)

where ${𝐅}_{i}$ is the feature representation of the *i*-th feature source, ${𝐠}_{i}$ is the gating weight vector for the *i*-th feature source, σ is the Sigmoid function, defined as $\sigma (x)=\frac{1}{1+e^{-x}}$, which constrains the values of ${𝐠}_{i}$ to the range [0, 1], and ⊙ denotes element-wise multiplication, meaning each dimension of the gate weight $\sigma ({𝐠}_{i})$ is applied to the corresponding dimension in ${𝐅}_{i}$.

Feature Fusion: To construct a unified feature representation, all gated features are aggregated via element-wise summation followed by averaging. This strategy ensures numerical stability and maintains scale consistency with the original feature distributions, facilitating effective integration of information from multiple sources. The mathematical formulation of the fusion process is defined as follows:

$${𝐅}_{\text{fused}}=\frac{1}{N}\sum_{i=1}^{N}{\tilde{𝐅}}_{i}$$ (5)

#### Neural network layer.

Following feature fusion, the integrated representation is passed through a hierarchical neural network architecture designed to capture semantic, structural, and local-functional dependencies. This architecture comprises a BiLSTM module, a parallel set of 1D convolutional layers (CNN), and a feed-forward network (FFN). Notably, the structural parameters governing these components—including the hidden size and number of BiLSTM layers, the kernel sizes and output channels of the CNN, and the dimensionality of the FFN—are not manually set but are instead optimized via a genetic algorithm (GA) [34]. This evolutionary strategy ensures that the model architecture is well adapted to the inherent complexity of peptide sequence data.

The first component, BiLSTM, captures bidirectional contextual dependencies within amino acid sequences. While it does not explicitly model 3D structures, peptide function often depends on residue interactions that are distant in sequence but proximal in spatial conformation. BiLSTM facilitates the modeling of such long-range dependencies by enabling information to flow from both the N-terminus and C-terminus simultaneously, thereby supporting functional inference from the sequence context.

However, recurrent models may underemphasize short, contiguous motifs that are often functionally critical. To address this, the output of the BiLSTM is passed to a set of parallel 1D convolutional layers, whose kernel sizes are optimized via GA. Each kernel size focuses on a different resolution of local patterns: smaller kernels capture fine-grained residue motifs, while larger kernels detect broader regional features. ReLU activation [35] introduces non-linearity, and max-pooling reduces dimensionality while retaining key signals. The use of multiple kernel sizes in parallel ensures that essential spatial information is preserved across various receptive fields.

The resulting multi-scale feature maps are concatenated and fed into the FFN module, which abstracts higher-order interactions through a series of nonlinear transformations. The FFN is also subject to GA-based tuning, particularly with respect to layer width and normalization strategy. To ensure training stability and consistency in feature representation across all sequence positions, the FFN incorporates additive layer normalization and residual connections, inspired by Transformer architectures. This design facilitates more effective gradient propagation and preserves subtle sequence information that might otherwise be attenuated in deeper networks.

#### Classification layer.

The classification layer is implemented as a multi-layer perceptron (MLP) composed of several fully connected layers that progressively reduce the dimensionality of the extracted features while capturing higher-order interactions. Each hidden layer is followed by a ReLU activation to introduce non-linearity and enhance model expressiveness. The final output layer consists of 21 neurons, corresponding to the 21 predefined peptide functional categories. A sigmoid activation is applied to each output unit to produce independent probabilities ranging from 0 to 1. During inference, a probability threshold of 0.5 is used: if the predicted probability for a category exceeds this threshold, the corresponding function is assigned to the peptide, indicating potential functional activity related to that category.

#### Marginal focal dice loss.

To address the challenges of class imbalance and prediction uncertainty, we design MFDL that integrates two complementary components. First, it incorporates the principles of LDAM to assign larger margins to minority classes, encouraging the model to better recognize underrepresented functions. Second, it further integrates the MLFDL to emphasize hard-to-classify samples, thereby enhancing robustness and reducing overconfidence in easy predictions. The complete formulation of MFDL is presented in the following components.

**Class weighting.** To account for label imbalance, we define a class-specific weight *w*_*c*_ for each functional category:

$$w_{c}=\left\{ \begin{array}{cc} \frac{1}{\sqrt{N_{c}}}\times \frac{M_{\text{max}}}{\max\left(\frac{1}{\sqrt{N_{\text{maj}}}},\frac{1}{\sqrt{N_{\text{min}}}}\right)}, & \text{if using class weights}\\ 1, & \text{otherwise} \end{array}\right.$$ (6)

where *N*_*c*_ is the number of samples in class *c*, $N_{\text{maj}}$ is the number of samples in the majority class, $N_{\text{min}}$ is the number of samples in the minority class, $M_{\text{max}}$ is the maximum margin. When class weights are applied, each class is assigned a weight calculated as the square root of the inverse of the number of samples in that class. This ensures that classes with fewer samples receive higher weights, giving them greater importance during training. The calculated weights are then scaled relative to the maximum weight among the classes, ensuring proportional balance. If class weights are not applied, all classes are treated equally by assigning them a uniform weight of 1.

**Margin adjustment.** The predicted logits are adjusted by a margin *m*_*c*_ based on the class:

$$m_{c}=\left\{ \begin{array}{cc} \frac{1}{\sqrt{w_{\text{min}}}}\times \frac{M_{\text{max}}}{\max\left(\frac{1}{\sqrt{w_{\text{maj}}}},\frac{1}{\sqrt{w_{\text{min}}}}\right)}, & \text{if }y_{i}=1\\ \frac{1}{\sqrt{w_{\text{maj}}}}\times \frac{M_{\text{max}}}{\max\left(\frac{1}{\sqrt{w_{\text{maj}}}},\frac{1}{\sqrt{w_{\text{min}}}}\right)}, & \text{if }y_{i}=0 \end{array}\right.$$ (7)

$${\hat{y}}_{i}^{\text{marginal}}={\hat{y}}_{i}-m_{c}$$ (8)

where $y_{i}\in \{0,1\}$ denotes the ground truth label for the *i*-th sample, ${\hat{y}}_{i}$ represents the predicted logit for the *i*-th sample, ${\hat{y}}_{i}^{\text{marginal}}$ is the predicted value after marginal adjustment, *m*_*c*_ is the margin for class *c*, $w_{\text{maj}}$ is the weight of majority class samples, $w_{\text{min}}$ is the weight of minority class samples. This formula adjusts the predicted logit ${\hat{y}}_{i}$ during prediction by subtracting a margin *m*_*c*_ based on the respective weights of majority and minority classes. This operation shifts the decision boundary in favor of minority classes by introducing a larger margin.

**Probability computation.** We apply the sigmoid function to obtain probability scores:

$$p_{i}=\sigma \left({\hat{y}}_{i}^{\text{marginal}}\right)$$ (9)

where $\sigma (x)=\frac{1}{1+e^{-x}}$ is the sigmoid function. By applying the Sigmoid function to the margin-adjusted ${\hat{y}}_{i}^{\text{marginal}}$, any real number can be mapped to the interval (0,1), yielding an interpretable probability value *p*_*i*_. This maps logits to interpretable probabilities while preserving the margin adjustments.

**Focal dice loss components.** To emphasize difficult samples and prevent gradient vanishing, we use clipped, focal-scaled Dice components for both positive and negative samples.

$$p_{i}^{\text{pos}}=\left\{ \begin{array}{cc} \min(p_{i}+{\text{clip}}_{\text{pos}},1)\cdot p_{i}, & \text{if }y_{i}=1\\ 0, & \text{otherwise} \end{array}\right.$$ (10)

$${\text{Numerator}}_{\text{pos}}=\sum_{i=1}^{N}p_{i}^{\text{pos}}y_{i}$$ (11)

$${\text{Denominator}}_{\text{pos}}=\sum_{i=1}^{N}\left((p_{i}^{\text{pos}}{)}^{p_{\text{pos}}}+y_{i}^{p_{\text{pos}}}\right)$$ (11)

$${ℒ}_{\text{pos}}=1-\frac{2\cdot {\text{Numerator}}_{\text{pos}}}{{\text{Denominator}}_{\text{pos}}}$$ (12)

$$p_{i}^{\text{neg}}=\left\{ \begin{array}{cc} \min((1-p_{i})+{\text{clip}}_{\text{neg}},1)\cdot (1-p_{i}), & \text{if }y_{i}=0\\ 0, & \text{otherwise} \end{array}\right.$$ (13)

$${\text{Numerator}}_{\text{neg}}=\sum_{i=1}^{N}p_{i}^{\text{neg}}(1-y_{i})$$ (14)

$${\text{Denominator}}_{\text{neg}}=\sum_{i=1}^{N}\left((p_{i}^{\text{neg}}{)}^{p_{\text{neg}}}+(1-y_{i}{)}^{p_{\text{neg}}}\right)$$ (15)

$${ℒ}_{\text{neg}}=1-\frac{2\cdot {\text{Numerator}}_{\text{neg}}}{{\text{Denominator}}_{\text{neg}}}$$ (16)

where $p_{\text{pos}}$ and $p_{\text{neg}}$ are the focal loss exponents for positive and negative samples, respectively, ${\text{clip}}_{\text{pos}}$ and ${\text{clip}}_{\text{neg}}$ are the clipping thresholds for positive and negative predictions, $p_{i}^{\text{pos}}$ and $p_{i}^{\text{neg}}$ represents the adjusted probability value when the *i*-th sample is a positive sample and negative sample, Numerator represents the part where positive or negative samples are predicted correctly, while Denominator represents the part where positive or negative samples are predicted incorrectly. These formulas adjust the predicted probabilities for positive and negative samples and compute the loss based on the adjusted probabilities. This approach enables the model to balance the contributions of positive and negative samples during training, avoiding gradient instability caused by predicted probabilities being too close to 0 or 1. Additionally, it enhances the accuracy of both minority and majority class predictions in imbalanced datasets, thereby improving overall classification performance.

**Final loss composition.** The final loss is computed by combining the positive and negative components:

$$ℒ=\alpha \cdot {ℒ}_{\text{pos}}+(1-\alpha )\cdot {ℒ}_{\text{neg}}$$ (17)

And reweighted by sample-specific class weights:

$${ℒ}_{\text{weighted}}=\sum_{i=1}^{N}w_{y_{i}}\cdot {ℒ}_{i}$$ (18)

where ${ℒ}_{i}$ is the loss for the *i*-th sample. This formula alleviates the class imbalance problem by assigning class-specific weights to the loss of each sample.

Finally, the reduction method (mean, sum, or none) determines how losses are aggregated:

$${ℒ}_{\text{final}}=\left\{ \begin{array}{cc} \frac{1}{N}\sum_{i=1}^{N}{ℒ}_{\text{weighted},i}, & \text{if reduction is mean}\\ \sum_{i=1}^{N}{ℒ}_{\text{weighted},i}, & \text{if reduction is sum}\\ {ℒ}_{\text{weighted}}, & \text{if reduction is none} \end{array}\right.$$ (19)

This formulation allows MFDL to flexibly integrate class imbalance correction, margin adjustment, and difficulty-aware learning into a unified objective function.

### Performance metrics

We used five commonly used evaluation metrics to assess the performance of our model, namely Precision, Coverage, Accuracy, Absolute true, Absolute false, F1-score and Matthews Correlation Coefficient(MCC). Their mathematical expressions are as follows:

$$\text{Precision}=\frac{1}{N}\sum_{i=1}^{N}\frac{\left||L_{i}\cap L_{i}^{*}|\right|}{\left||L_{i}^{*}|\right|}$$ (21)

$$\text{Coverage}=\frac{1}{N}\sum_{i=1}^{N}\frac{\left||L_{i}\cap L_{i}^{*}|\right|}{\left||L_{i}|\right|}$$ (22)

$$\text{Accuracy}=\frac{1}{N}\sum_{i=1}^{N}\frac{\left||L_{i}\cap L_{i}^{*}|\right|}{\left||L_{i}\cup L_{i}^{*}|\right|}$$ (23)

$$\text{Absolute True}=\frac{1}{N}\sum_{i=1}^{N}\Delta (L_{i},L_{i}^{*})$$ (24)

$$\text{Absolute False}=\frac{\sum_{i=1}^{N}||L_{i}\cup L_{i}^{*}||-||L_{i}\cap L_{i}^{*}||}{M}$$ (25)

$$\text{F1-score}=\frac{2\cdot TP}{2\cdot TP+FP+FN}$$ (25)

$$\text{MCC}=\frac{TP\cdot TN-FP\cdot FN}{\sqrt{\left(TP+FP)(TP+FN)(TN+FP)(TN+FN\right)}}$$ (26)

$$\Delta (L_{i},L_{i}^{*})=\left\{ \begin{array}{cc} 1, & \text{if }L_{i}^{*}\text{ is identical to }L_{i}\\ 0, & \text{otherwise} \end{array}\right.$$ (27)

where *N* is the total number of peptide sequences in the dataset, *M* is the total number of label types in the label types, ‖ ‖ represents the operation that counts the number of elements, *L*_*i*_ denotes the subset with true labels for the *i*-th sample, $L_{i}^{*}$ denotes the subset with the labels predicted for the *i*-th sample, $\Delta (L_{i},L_{i}^{*})$ is 1 if $L_{i}^{*}$ is identical to *L*_*i*_, and 0 otherwise, *TP* is the number of true positives, *FP* is the number of false positives, *TN* is the number of true negatives and *FN* is the number of false negatives.
